# Physical Literacy-Focused Education Improves Fitness Markers in Preadolescents: Implications for School-Based Health Promotion

**DOI:** 10.3390/healthcare14050695

**Published:** 2026-03-09

**Authors:** Petra Rajkovic Vuletic, Tomislav Pranjic, Barbara Gilic Skugor, Blaž Lešnik, Damir Sekulic, Dorica Šajber

**Affiliations:** 1 Faculty of Kinesiology, University of Split, 21000 Split, Croatia; petra.rajkovic@kifst.eu (P.R.V.); tomislav.pranjic@kifst.eu (T.P.); damir.sekulic@kifst.eu (D.S.); 2 Faculty of Kinesiology, University of Zagreb, 10000 Zagreb, Croatia; 3 Faculty of Sport, University of Ljubljana, 10000 Ljubljana, Slovenia; blaz.lesnik@fsp.uni-lj.si (B.L.); dorica.sajber@fsp.uni-lj.si (D.Š.)

**Keywords:** motor activity, pediatrics, quasi-experimental studies, health promotion

## Abstract

**Highlights:**

**What are the main findings?**

- A physical literacy-based intervention led to small, but significant, improvements in specific fitness domains among preadolescent children, particularly jumping power and abdominal strength.
- The intervention was successfully implemented within regular physical education classes, requiring no additional resources or time, making it a real-world, scalable model for schools.

**What are the implications of the main findings?**

- Enhancing physical fitness in preadolescents can lay a foundation for lifelong physical activity, potentially reducing the future burden of chronic non-communicable diseases.
- The applied model is suitable for public health programs in schools, especially in low-resource settings.

**Abstract:**

Background/Objectives: Physical literacy (PL) is globally recognized as a foundational determinant of health status in children, but the effects of interventions based on PL were rarely studied in preadolescent children. The aim of this quasi-experimental, school-based study was to evaluate the potential effects of a PL intervention delivered during regular school hours on physical fitness (PF), physical literacy (PL), and physical activity (PA) in children aged 9 to 11 years from southern Croatia. Methods: Participants were 125 preadolescents (57 girls), and were grouped into a control group (C = 70), and an experimental group (E, n = 55). The E group participated in a specific PL-focused education, integrated into regular physical education (PE) lessons as a 5–6 min substitute for standard PE content, over 12 weeks (36 PE classes in total). The C group participated in the standard PE curriculum. A pre–post–retention design was used, and the observed variables included anthropometrics (height, weight, BMI), PF (jumping power, abdominal strength, upper body strength, flexibility, mobility, and cardiovascular endurance), PL (evaluated by the PLAYself questionnaire), and indirectly measured PA (estimated via the PAQ-C questionnaire). Multivariate (MANOVA and univariate (ANOVA) analyses of variance for repeated measurements were used with time, gender, and group as the main effects, including their interactions. Results: MANOVA calculated for a set of PF variables revealed significant main effects for time (F = 1361, *p* < 0.001) and a significant time × group interaction (F = 2.98, *p* < 0.05). Univariate ANOVA indicated specific intervention effects for jumping power and abdominal strength, favoring the E group. No significant differential effects were observed for PL or PA. Conclusions: The study demonstrated small, but statistically significant, improvements in PF indicators among children exposed to the PL-based intervention. Notably, the intervention was conducted in authentic school settings using standard resources, supporting the ecological validity and real-world applicability of the findings.

## 1. Introduction

It is widely accepted that physical fitness (PF) serves as one of the main indicators of health in children and adolescents [1,2,3,4]. In brief, optimal PF during childhood and adolescence represents a fundamental basis for the adoption of healthy lifestyle habits and is an important factor reflecting the health status of the individual [2,5]. Supportively, studies indicate a strong association between high levels of PF in youth and better health status in adulthood [2,5]. Furthermore, maintaining adequate PF in youth significantly reduces the risks of overweight/obesity, various cardiovascular diseases, and type II diabetes [6,7]. Findings from research on mental health suggest a positive relationship between PF and general mental well-being in children and youth [8]. Finally, studies repeatedly reported better quality of life in children with better PF, with extensive evidence also highlighting a significant relationship between PF and academic performance [9,10]. Among the components of PF, cardiorespiratory fitness and muscular strength have been identified as key factors with a strong and consistent impact on health [11]. However, despite the robust body of evidence supporting the importance of PF, there are significant indicators of a continued decline in PF levels among children and adolescents [12,13].

Regular physical activity (PA) is a fundamental prerequisite for achieving and maintaining optimal levels of PF and is therefore considered a key factor in preserving and improving the health of children and adolescents [14]. Studies conducted on youth populations confirm that PA is the primary driver for the development of essential PF components, such as aerobic endurance and muscular strength, with significant effects particularly evident in high-intensity physical activities [15]. At the same time, higher levels of PF enable individuals to engage in more intense and prolonged PA, as they exhibit greater load tolerance [15,16,17,18]. Therefore, it is widely accepted that encouraging diverse forms of PA throughout development is crucial for fostering overall PF and ensuring long-term health maintenance [19]. However, existing research indicates that childhood and adolescence represent critical periods for establishing these positive interrelations [14,20,21]. Therefore, in order to achieve optimal health benefits, programs and interventions targeting children should simultaneously promote both PA and PF.

Due to the previously outlined challenges, research increasingly focuses on identifying effective methods to enhance levels of PA and improve PF [22,23,24]. In general, there is a general consensus that increasing PA positively influences various dimensions of PF [25,26,27]. For instance, a US study reported a positive correlation between PA levels and all measures of PF among children aged 9 to 11 years [25]. Likewise, research on Spanish children aged 8 to 12 years also confirmed that higher PA levels are associated with better PF outcomes [26]. Furthermore, Danish authors studied children aged 8 to 10 years and demonstrated that those children who engage in sports and exhibit higher PA levels show superior cardiorespiratory function, sprint times, motor coordination, and better body composition compared to peers with lower PA levels [27]. A longitudinal study conducted in Estonia, which followed children from preschool through the end of grade five (up to 11.5 years of age), also confirmed the positive effects of regular participation in sports activities on health and PF [28]. More accurately, the study revealed that 73% of children who remained physically active achieved optimal PF levels, compared to only 24% among inactive peers [28]. Additionally, a recent study from Poland highlighted the positive effects of increasing PE class hours (and consequently increasing PA levels) on PF in children aged 11 to 12 years [29]. However, growing evidence suggests that merely increasing PA does not necessarily lead to improvements in PF among children. Therefore, it is recommended to focus on promoting those forms and intensities of PA that directly contribute to enhancing PF levels [15,30,31,32]. With regard to all that has been said, the concept of physical literacy has gained significant attention over the past decade as an integrative and comprehensive framework for understanding and promoting their development.

Physical literacy (PL) can be defined as an individual’s ability to demonstrate the physical competence, confidence and motivation to engage in PA, along with an understanding of its health benefits, in order to develop lifelong habits of active living [33,34]. Studies confirmed that interventions aimed at developing PL result in significant improvements in components of PF and simultaneously increase levels of PA among children and adolescents, thereby supporting their overall developmental goals [35,36]. For instance, pedagogical intervention demonstrated significant improvements in various fitness capacities in children aged 8 to 12 years [37]. Further, a six-month program targeting PL development showed substantial gains in PF among children aged 5 to 12 years [38]. Moreover, a twelve-week intervention designed to enhance PL resulted in significant increases in both PA and PF levels among children aged 9 to 11 years [39]. A five-month program based on structured and active play reduced sedentary behavior, and increased PA levels among children aged 6 to 9 years [40]. In contrast, an extracurricular intervention focused on creativity, conducted over five months, did not yield statistically significant changes in PF among children aged 8 to 11 years [41]. However, the intervention integrating various dimensions of PL in children aged 8 to 11 years demonstrated statistically significant improvements in PF [42].

Collectively, it is widely accepted that PL deserves particular attention in global efforts aiming at simultaneous improvement of PF and PA in children. Specifically, most of the studies which targeted improvement of PL as a way of improving PF and PA reported positive effects [35]. Nevertheless, it is crucial to highlight several limitations of previous studies. First, the majority of previous studies did not include a control group (i.e., the improvements observed may result from external factors such as maturation and/or seasonal effects). Second, it seems that none of the studies done so far have conducted follow-up assessments, which prevents evaluation of the long-term sustainability of effects, making it unclear whether observed changes persist beyond the immediate post-intervention period [35,36]. Furthermore, the assessment of PF in most studies relied on a limited number of tests, often focusing predominantly on cardiorespiratory fitness indices, which may not comprehensively capture the multifaceted nature of PF [35,36]. Therefore, this study aimed to evaluate the effects of a PL intervention on changes in PL, indirectly measured PA and various components of physical PF in Croatian preadolescents, aged 9 to 11 years. The hypothesis posits that the PL intervention incorporated in the regular PE curriculum will lead to increased PA, better PL, and positive changes in PF components in preadolescent children.

## 2. Materials and Methods

### 2.1. Participants

A total of 125 preadolescents (68 boys and 57 girls) from southern Croatia participated in this study. At the time of assessment, they were 9–11 years old, and were enrolled in the 3rd (n = 74) or 4th (n = 51) grade of elementary school, healthy, and regularly attending physical education (PE) classes. Exclusion criteria included illness or locomotor injury. Written informed consent was obtained from parents/guardians after they were briefed on the study’s aims and procedures by the research team. The study protocol was approved by the Ethics Committee of the Faculty of Kinesiology, University of Zagreb.

**Figure 1:**
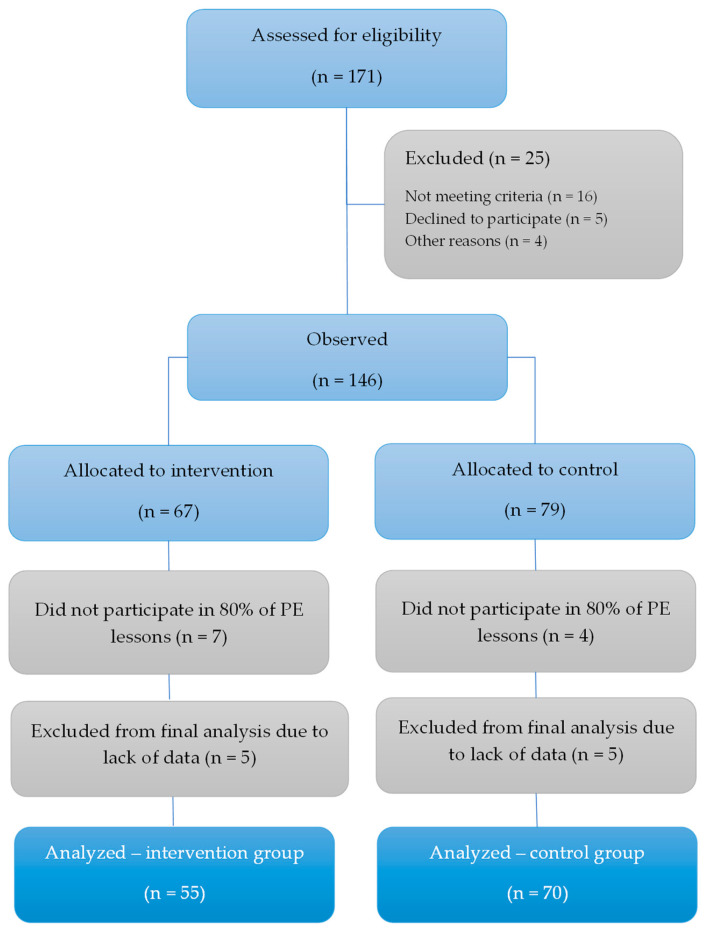
Flow diagram of the investigation.

Figure 1 presents a flow diagram of the study.

The participants were grouped into an experimental/intervention group (E, n = 55) and a control group (C, n = 70). Since intervention was delivered as a part of PE, we applied a quasi-experimental design. In particular, intact classes (rather than individual students) were assigned to either the E (two classes) or the C (two classes). Because participants were nested within classes, intraclass correlation coefficients (ICCs) were calculated for the primary outcome variables to estimate the magnitude of clustering. The observed ICC values for fitness variables were low (ICC = 0.01–0.04), suggesting that only 2–4% of the total variance was attributable to class-level differences. This magnitude is below commonly cited thresholds indicating meaningful clustering effects in school-based research (e.g., ICC ≥ 0.05) [43]. Therefore, multilevel modeling was not deemed necessary.

### 2.2. Variables

Besides gender (female/male), and age (years), key outcomes included anthropometric variables (body height, body mass, and calculated body mass index), PF, PL, and indirectly estimated PA. An experienced technician with substantial expertise in assessments performed the anthropometric measurements. PF tests were performed by PE teachers, with five examiners each evaluating an entire group simultaneously. All assessors were experienced, had undergone study-specific training, and were familiar with the protocols, which align with routine Croatian PE fitness testing practices.

The PL was measured using the Croatian version of the PLAYself questionnaire, a component of the PLAY tools (PLAYfun, PLAYbasic, PLAYself, PLAYparent, PLAYcoach, PLAYinventory [44]. Based on self-evaluation protocol, it comprises four subdomains: (i) environment, evaluating confidence in movement across various settings (e.g., gym activities, water, snow, and ice); (ii) self-report of PL, measuring affective and cognitive aspects (e.g., motivation, confidence, self-esteem) that influence self-efficacy and PA participation (i.e., “It does not take me long to learn new skills, sports or activities”); (iii) relative ranking of literacy (including literacy, numeracy, and physical literacy subdomains), assessing literacy valuation in contexts like school, home, and peers; and (iv) fitness status, determined by the question “My fitness level is good enough to allow me to participate in all the activities I choose” (excluded from the final score). The overall score is calculated as the average from the first three subdomains across 27 items, with a maximum of 100 indicating strong self-perception.

In order to evaluate the PA, we used the Croatian version of the ”Physical Activity Questionnaire for Older Children” (PAQ-C) [45]. This 9-item instrument uses a 5-point scale to compute a mean score reflecting habitual activity (range: 1 = minimal; 5 = maximal), with sample items including “What did you do during long breaks last week (excluding snacks)?” and “Daily physical activity frequency last week.” Questionnaires were completed online in classroom groups of ≤10 children, with an examiner available for support but positioned to prevent viewing responses, thereby maintaining confidentiality.

Body height (BH) was measured using a stadiometer, with participants standing barefoot on a firm, level surface in an upright posture and the head aligned to the Frankfurt horizontal plane (gaze straight ahead). Measurements were recorded to 0.1 cm precision. Body mass (BM) was assessed via a digital scale, wearing shorts and a sports shirt, and results reported in kilograms to 0.1 kg accuracy. Body mass index (BMI) was calculated by dividing participants’ body mass (in kg) by their squared body height (in meters).

PF was assessed using tests from the FitnessGram battery (body height and mass, aerobic (cardiovascular) endurance, flexibility, torso extensor strength, abdominal and upper body strength), and standing broad jump [46]. Aerobic endurance was evaluated using the 15 m multistage fitness test (beep test). The test comprises running between 15 m divided lines. Participants have to step on the line on each beep and turn promptly, continuing until they are unable to reach the line before the second beep; scores reflect completed levels. Abdominal strength/endurance was measured by the sit-up test (sit-ups), where participants performed maximal torso lifts (up to 75), scored as correctly executed repetitions. Torso extensor strength/flexibility was assessed via the torso lift test: Participants raised the cervical/thoracic spine controllably to maximum extension, maintaining neutral head alignment with the spine. Upper body strength/endurance used the push-up test: From a plank position (palms under shoulders, legs extended), participants lowered until elbows reached 90° (upper arms parallel to floor) then extended fully, keeping the body straight; maximum correct repetitions were recorded. Lower back/hamstring flexibility was tested with the sit-and-reach: Seated with legs extended against a box, participants reached maximally forward (palms down), holding 2+ seconds; the best average of three trials was used. Jumping power (explosive strength) was assessed by the standing long jump (broad jump): Barefoot from a starting line, participants jumped maximally forward onto a standardized mat (ELAN, Begunje, Slovenia); the best of three attempts in cm was used as a final result.

### 2.3. Intervention and Protocol of the Study

**Figure 2:**
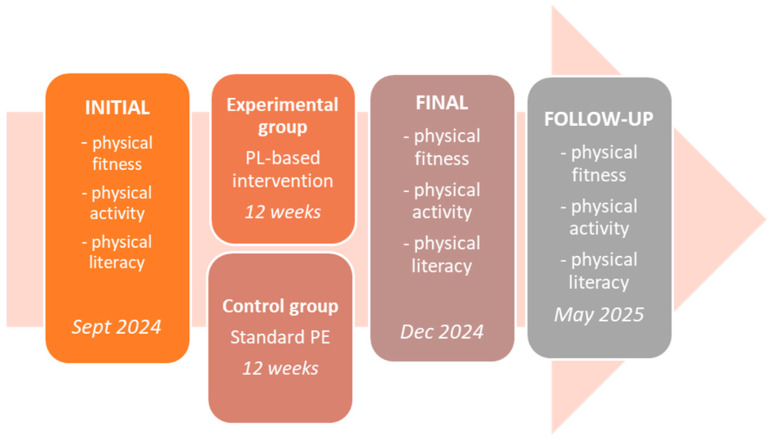
Study protocol.

An overview of the study timeline is presented in Figure 2.

The educational intervention, focused on PL, was implemented within the existing PE schedule for the experimental group over a 12-week period. During this time, students received three PE sessions weekly (36 lessons in total). The intervention incorporated 12 short, original educational videos (3–4 min each), each addressing a key topic related to PL or physical health. The video content was organized into the following themes: two videos covered core PL principles (e.g., movement motivation, confidence, and understanding), three focused on cardiorespiratory fitness, five targeted motor abilities (strength, power, coordination, flexibility), and two addressed general health habits, including nutrition. Each video was shown three times to reinforce learning and ensure knowledge retention (please see Supplementary Materials for video education, Video S1). A short overview of the video contents is presented in Table 1.

Concise, focused videos are known to be especially effective for maintaining children’s attention and promoting engagement, especially in the situations when videos are followed by guided discussions, such as the one we applied in the study [47]. The development of the video content was carried out in consultation with both PE specialists and medical professionals to ensure accuracy, relevance, and age-appropriateness. This collaborative approach helped align the content with curricular goals while also addressing key health promotion messages. As a result, the videos reflected evidence-based practices from both educational and health perspectives. Notably, similar approaches were found to be successful among adolescents during COVID-19 disruptions to traditional schooling [48]. Also, this PL-educational program has recently shown promising results in the context of maintaining PA levels during the school year in early school age children, and the authors noted the necessity of evaluating its effectiveness for other important health related indices, particularly PF status [49].

With regard to differences between the experimental and control group, it should be stated that the Croatian PE practices (45 min in total) typically consist of a warm-up (5–10 min), main activity block (30 min), and a closing section (5–10 min). The experimental educational content was delivered during the final segment of each class. As such, the motor content of PE lessons remained consistent across both study groups. To integrate the intervention without disrupting the core PE structure, PL-education replaced <7 min of standard activity. The control group also received 36 PE lessons over the same period, but followed the national curriculum without additional PL-based content. Intervention structure is provided in Table 2, emphasizing the most important differences between the control and experimental group.

This research is a continuation of the previous research in the field [48,49]. However, this study extends previous works conducted within the same educational framework (PL-based intervention) but differs in several important methodological and conceptual aspects [49]. Whereas recent research investigated the effects of the PL intervention on PA and body composition, the current study focuses specifically on PF outcomes, including muscular strength, explosive power, flexibility, and aerobic endurance. Next, this study included younger participants, and/or observed a larger sample than previous ones [48,49]. Finally, previous studies used univariate analysis exclusively, while herein, multivariate statistical procedures were applied (please see Statistics below) [48,49].

### 2.4. Statistics

The Kolmogorov–Smirnov test was applied to evaluate the normality of distributions for all variables. Descriptive statistics included the calculation of means and standard deviations (for continuous variables), and counts and percentages (for categorical variables).

To control for potential inflation of Type I error due to multiple outcomes, a hierarchical analytical strategy was applied. Specifically, a multivariate repeated measures analysis of variance (MANOVA) was first conducted for the full set of PF variables to test for overall time and interaction effects while accounting for intercorrelations among dependent variables. Since the multivariate test reached statistical significance in the next phase, we applied a 2 (Group: experimental vs. control) × 2 (Gender: boys vs. girls) × 3 (Time: pre-, post-, and retention-test) mixed-design analysis of variance (ANOVA) with repeated measures on the time factor for each dependent variable. Post hoc comparisons were conducted for variables showing significant ANOVA effects. Additionally, Bonferroni correction was applied to post hoc comparisons where appropriate. This stepwise approach reduces the likelihood of Type I error inflation by limiting secondary analyses to statistically justified cases. Homogeneity of covariance matrices was tested using Box’s M test, and Levene’s test was used to assess the equality of error variances. Cell sizes were reviewed to ensure approximate balance across conditions.

Initially, pubertal timing was estimated for all children using peak height velocity (PHV), as described by Moore et al. (PHV = −7.999994 + (0.0036124 × age (yrs.) × height (cm)) [50]. In order to control for the possible influence of maturity status on changes which occurred during the study course, the PHV was included as a covariate in ANOVA and MANOVA calculations.

Effect sizes for ANOVA main and interaction effects are reported as partial eta squared (η^2^), interpreted as follows: small (0.01–0.06), medium (0.061–0.14), and large (≥0.141). When significant effects were found, post hoc analyses were performed using Scheffé’s test.

To reduce the risk of Type I error due to multiple comparisons, Bonferroni correction was applied as appropriate.

All statistical analyses were conducted using Statistica v14.5 (TIBCO Inc., Palo Alto, CA, USA), with the level of statistical significance set at *p* < 0.05.

## 3. Results

MANOVA calculated for PF variables indicated significant main effects for time (large ES). Also, significant interaction effects were found for “time × group” (medium ES), indicating differential changes in physical fitness variables in study groups over the study course (Table 3).

Table 4 presents the results of the ANOVA, with corresponding effect size values. Significant main effects for “time” were found for all variables, specifically: body height (large ES), body mass (medium ES), BMI (medium ES), all PF tests broad jump (medium ES), push-ups (medium ES), sit-ups (large ES), torso lift (medium ES), sit-and-reach (large ES), multistage fitness test (small ES), physical literacy (medium ES), and PA (small ES). When observing the main effect for “gender”, the F-test reached statistical significance for sit-and-reach (large ES), multistage fitness test (medium ES), and PA (medium ES). With regard to the “time × gender” interaction effect, the F-test was significant only for body height (small ES). The interaction effect for “time × group” was significant for broad jump (small ES) and sit-ups (small ES), indicating differential changes in the control and experimental group during the study course.

Table 5 presents descriptive statistics for the total sample of participants at initial, final- and follow-up testing, with the significance of the ANOVA post hoc differences. Specifically, analyses indicated an increase in the body height and body mass throughout the study course, with significant post hoc (*p* < 0.05) differences between all three measurements (initial-, final, and follow-up testing). The BMI values increased from initial to final, and decreased until follow-up measurement, with significant post hoc differences between the first two measurements. The performance in broad jump and sit-ups improved throughout the study course, with significant post hoc differences between initial and follow-up measurement. Changes in the push-ups test varied, with a decrease from the initial to final measurement and an increase from final to follow-up testing (significant post hoc differences between final and follow-up). On the other hand, children improved their performance in the torso lift between initial and final testing, but results were worsened between final and follow-up (significant post hoc differences between initial and final measurement). Sit-and-reach performance slightly improved in the first part of the study (significant post hoc differences between the initial and remaining two measurements). The results of the multistage fitness test improved during the study course, with significant post hoc differences between initial and follow-up testing. The PL improved during the study course (significant post hoc differences between initial and follow-up measurement). Finally, results showed significantly higher PA at the follow-up measurement than in the initial testing. Relative changes which occurred during the study course for the total sample, as well as for the control and experimental groups, are presented in the Supplementary Figure S1.

**Figure 3:**
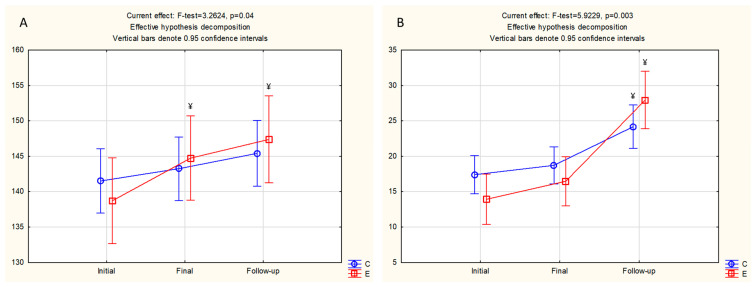
Descriptive statistics (data are presented as means + 95% confidence intervals) for study control (C), and experimental (E) group ((**A**)–broad jump, (**B**)—sit-ups) and significant post hoc differences for variables with significant ANOVA time × group interaction effects (^¥^ significantly different from initial measurement).

Figure 3 presents the descriptive statistics and significance of the post hoc differences, for those variables where ANOVA evidenced a significant “time × group” interaction effect, and consequently indicated differential changes in studied variables in the control and experimental group. For the broad jump, the experimental group significantly improved performance, with significant differences between initial and final measurement, and initial and follow-up measurement. Simultaneously, no significant changes were evidenced in the control group for the same performance (Figure 3A). When analyzing for sit-ups (Figure 3B), both groups improved their achievement during the study course, with significant differences between initial and follow-up measurement. However, the improvement was more evident in the experimental group than in the control group.

## 4. Discussion

Following are the most important findings indicated by the results. First, PL intervention resulted in small but statistically significant improvements in certain dimensions of PF, specifically power and strength capacities. Second, there was no significant effect of PL intervention on indirectly measured PA and affective and cognitive domains of the PL. Therefore, our initial study hypothesis can be partially accepted.

### 4.1. Effects of Physical Literacy-Based Intervention on Physical Fitness

Our study demonstrated the benefits of PL-based intervention in improving children’s PF, specifically lower-body explosive strength (jumping power capacity) and the dynamic muscle strength of abdominal muscles. In exploring the potential reasons for this improvement, several factors emerge as the most plausible. First, it is likely that the intervention, which included brief, clearly structured educational content on PL, contributed to bettering participants’ knowledge about physical fitness and training, which resulted in positive attitudes and stronger intentions to engage in PA. Specifically, the educational video contents, such as “What is strength and how do we train it?”, likely stimulated greater engagement in strength-related tasks (e.g., squats, sit-ups, core exercises) during PE classes, but also in free time. This likely resulted in a greater accumulation of effective repetitions in the experimental group, compared to the control group.

It is important to highlight that both the standing broad jump and sit-up tests are tasks where improvements can be achieved relatively quickly through increased effort (in the case of sit-ups) and minor technical adjustments (e.g., improved arm swing, take-off preparation, execution rhythm in the case of the broad jump), without requiring long-term, sport-specific training [51,52]. Encouragingly, this pattern of effects aligns with findings from a meta-analysis that included 48 intervention studies conducted with children and adolescents aged approximately 6 to 18 years, all focused on the development of PL [35]. In general, it was found that interventions similar to the one applied in our study produce statistically significant effects in PF and motor skills [35]. Additionally, a study implementing PL interventions in children aged 7 to 9 years also reported significant improvements in PF components, further supporting the findings of our research [53].

Another possible explanation of the achieved positive effects deserves attention. Namely, it is possible that interventions incorporating education based on PL significantly enhances children’s sense of self-efficacy, fostering beliefs such as “I can do this” and “I know how to do this.” This contributes to greater effort, persistence, and improved performance in motor tasks [54,55,56,57]. Specifically, when high cognitive load occurs during physical exercising, it often reduces perceived self-efficacy. Altogether, it partially mediates declines in performance, and reduces the participation in physical exercising itself [55]. As a result, children with high self-efficacy are more likely to engage in PA [56]. Therefore, it can be said that high physical self-efficacy encourages engagement, greater effort, and improved outcomes in fitness tests by enhancing understanding, perceived competence, enjoyment, and intrinsic motivation [54,58,59]. Taken together, all these factors (e.g., enhanced self-efficacy, reduced cognitive load, and improved technique) could lead to the obtained positive PF outcomes evidenced in the experimental group.

Although the PL-educational content included topics related to the importance of flexibility and aerobic (cardiovascular) endurance, as well as strategies for their development, no significant effects were observed in these components of PF. The authors believe this may be explained by several factors. First, we cannot neglect the simple fact that, during the period of intervention, participants did not have a sufficient volume of continuous, moderate-to-vigorous-intensity activities necessary to stimulate cardiovascular adaptations that typically lead to improvements in aerobic endurance [60]. Most specifically, the intervention was applied in the period of three months, in unfavorable weather conditions autumn and winter period). As a result, it was hard to expect participants to participate in activities which would be suitable for the development of aerobic endurance, such as prolonged running, hiking, bicycling, and similar. Therefore, environmental conditions may have attenuated the potential impact of the intervention on cardiorespiratory fitness outcomes. It is important to note that existing research suggests that aerobic endurance improvements require a longer, structurally adapted, and sufficiently intense intervention, often involving several weeks of systematic training, while purely informational interventions, without a clear increase in training volume or load, frequently fail to produce measurable outcomes [61].

It is important to note that other studies examining the effects of interventions focused on PL, where authors also reported limited impact on aerobic endurance, often attributed to the short duration or educational emphasis of the programs [35]. Specifically, an afterschool PL intervention involving 15 min of fundamental motor skills (FMSs) and games over an 8-week period in children aged 5 to 12 years did not lead to improvements in aerobic endurance [62]. Similarly, a 12-week afterschool PL program incorporating games and skill-based activities in children aged 5 to 12 years showed no significant improvements in cardiorespiratory endurance [38]. Also, a 10-week PL program combining track and field games with functional movement skill exercises for children aged 8 to 12 years did not result in changes in aerobic endurance indicators [63].

Similar to our findings, previous studies focused on PL have also failed to demonstrate significant effects on flexibility. For example, the previously cited study by Chinese authors did not report any intervention-related improvements in flexibility measures [53]. Additionally, in a study comparing a sports-oriented elementary school with a traditional school, no effects on flexibility were found despite the higher volume of organized PA in the sports school [64]. This suggests that neither a more intensive nor a sport-enriched curriculum alone guarantees improvements in flexibility unless flexibility-specific content is systematically and purposefully included in the program [64]. There are several possible explanations for this. First, the pronounced longitudinal bone growth during this developmental period may partially obscure actual flexibility achievements, as most flexibility tests, including the one we have applied here (e.g., sit-and-reach), do not account for changes in skeletal proportions [65]. Second, flexibility as a component of PF is generally less appealing to children of this age group. In other words, it is unlikely that children will engage seriously in training activities aimed at improving flexibility.

### 4.2. Lack of Effects in Indirectly Measured Physical Activity

The PL intervention did not produce a significant effect on indirectly assessed PA. The first possible explanation for the absence of a significant effect on PA lies in the timing of the study itself (September–December–May). During the initial measurement period, children had not yet encountered intense academic obligations and had more free time available for spontaneous PA, such as outdoor play. The final measurement was conducted in December, during the winter months, when days are shorter and weather conditions colder, factors well-known to reduce overall PA in children by limiting opportunities for outdoor activities [66,67,68]. The follow-up measurement was conducted near the end of the school year, a period typically associated with heightened academic demands [66,67,68]. Supportively, a recent meta-analysis involving children aged 3 to 18 years confirms that winter is associated with a significant decline in PA and an increase in sedentary behavior [67]. Collectively, these factors likely equalized PA levels across groups and masked any specific effects of the intervention on PAQ-C scores.

Second, the lack of effects in PA can also be attributed to the limitations of indirect assessment using self-report questionnaires. Specifically, the PAQ-C, as a self-administered tool, assesses general levels of PA over the past seven days, focusing primarily on moderate-to-vigorous PA during daily routines, leisure time, and sports participation. This makes it a subjective measure, prone to recall bias [69,70]. Additionally, we cannot overlook the potential influence of social desirability bias. In self-report instruments such as the PAQ-C, where participants are asked about socially acceptable and expected behaviors (i.e., being engaged in higher levels of PA), children may tend to report more favorable behaviors than those actually performed (i.e., in our case, they will probably report higher PA). This tendency may lead to the overestimation of PA and may reduce the sensitivity of the questionnaire [71]. Studies have already shown that the PAQ-C may have weak correlations with objective measures of PA and may not be sensitive to changes resulting from specific types of physical training or fitness improvements [71,72]. This implies that an intervention which enhances specific motor abilities does not necessarily lead to detectable changes in the overall pattern or frequency of PA typically performed by children, which is what the PAQ-C is designed to measure. Indeed, a recent study evidenced favorable changes in PA as a result of PL intervention, when PA was objectively measured by accelerometers [49]. Collectively, the lack of significant effects in PA can be interpreted as a consequence of both the limitations of the measurement instrument and the influence of seasonal factors, rather than as evidence of the intervention’s failure to enhance overall PA in children.

### 4.3. Lack of Effects in Physical Literacy Facets

Although PL in children generally increased (see Results for details), the intervention did not result in significant differential effects on PL, specifically in the affective domain (motivation and confidence) and the cognitive domain (knowledge and understanding), as assessed by the PLAYself questionnaire. The most probable explanation for this outcome is that children in their self-assessments of PL primarily compared themselves to peers within their own group (i.e., children in the experimental group compared themselves to other children in the same group, and similarly in the control group). In this context, their perceived physical competence may not have changed in relative terms. A previous study assessing PL in children aged 9 to 11 years showed that the PLAYself questionnaire demonstrates good reliability and validity, but also revealed that children’s self-perceptions of PL are largely shaped through peer comparisons within the same social context, potentially “stabilizing” their responses in the absence of external reference points [44]. Under such circumstances, children rarely perceive personal progress unless they have the opportunity to compare themselves with other groups or against objective standards. A similar pattern of effects was reported in a recent experiment where authors analyzed the effects of the 8–9-week enriched PE intervention for children aged 9 to 11 years, and reported no statistically significant changes in the affective and cognitive domains as measured by the PLAYself questionnaire [42].

On the other hand, a 10-week PL program involving track and field games and fundamental motor skill exercises in children aged 8 to 12 years, assessed using another measurement tool (e.g., CAPL-2 questionnaire), led to significant improvements in the cognitive domain of PL [63]. Similarly, a study that implemented a 13-week PL intervention in children aged 9 to 12 years, combining classroom instruction with digital content during the regular school day and assessed using CAPL-2, also demonstrated statistically significant improvements in the cognitive domain [73]. Also, positive findings were found in a pilot study on active recess in preadolescents (8–12 years), which involved four weeks of short, structured, and content-focused activities, and reported significant improvements in both the affective and cognitive domains, also assessed by the CAPL-2 questionnaire [74].

Taken together, it seems that intervention studies that used the PLAYself questionnaire (the one we used herein) did not report significant changes in the affective and cognitive domains of PL following the intervention questionnaire [42]. In contrast, studies reporting improvements in these domains predominantly regularly used the CAPL-2 assessment tool [63,74]. This pattern raises the possibility that CAPL-2 may be more sensitive or better suited to capturing changes in motivation, confidence, knowledge, and understanding among children, particularly in the context of short-term interventions.

In addition to all that has been discussed, we must note that, although the PLAYself questionnaire has shown good reliability and validity in previous studies, it is generally known that self-assessment tools in children can be influenced by social desirability bias [44]. This means that children may answer in a way they think is expected by teachers or researchers, instead of reporting their true thoughts or abilities. Such responses may reduce the sensitivity of the questionnaire to detect small changes after an intervention. Nevertheless, in the present study, PLAYself was intentionally used due to its previously established psychometric robustness in Croatian children, including cultural and linguistic adaptation, and its feasibility for use in a school-based setting [44]. However, given the previously discussed improvements in PF, it seems reasonable to conclude that the PL intervention did lead to actual gains in PL, but that these changes were not adequately captured by the chosen measurement tool.

### 4.4. Implications of Results for School-Based Strategies and Health Promotions

Although the effects of the intervention were not large, they are still important when considered in the real school setting in which the program was carried out. For example, improvements in lower-body explosive strength are important because this type of strength supports everyday activities such as running, jumping, climbing stairs, and active play. Improvements in abdominal strength are also meaningful, as core muscles help maintain good posture, stabilize the body during movement, and may reduce the risk of injury. Importantly, these improvements were achieved without adding extra class time, increasing training intensity, or requiring additional equipment. Instead, they resulted from small changes within regular PE lessons.

The findings of this study reinforce the potential of school-based PL interventions as practical and effective strategies for promoting children’s PF. By embedding short, structured, PL-focused educational content into regular PE classes, the intervention led to significant improvements in certain components of PF. These outcomes highlight that even minimal, well-targeted modifications to existing PE curricula can yield measurable benefits. Importantly, the intervention required no additional resources or scheduling, making it highly feasible and scalable within standard school environments. From a public health perspective, integrating PL principles into schools may help establish the foundation for healthy movement habits, which are essential for the prevention of pediatric obesity, musculoskeletal issues, and cardiometabolic risk factors [75].

Moreover, the study underscores the ecological validity and real-world applicability of PL-based health promotion within the school setting. Conducted during regular school hours with regular PE teachers, the intervention mirrors the actual conditions under which health promotion strategies must operate, ensuring greater translational relevance. Therefore, our results actually underscore the opinion that schools should be considered vital platforms for preventive health measures, and that PL-based content represents a promising, cost-effective approach to improving children’s physical and mental well-being [76].

### 4.5. Limitations and Strengths

This study has several limitations that should be acknowledged. First, the allocation to intervention and control groups was done at the class level rather than through individual randomization. Although this approach is generally consistent with established practices in school-based cluster randomized trials designed to minimize contamination effects and preserve natural classroom structures, such an approach may result in residual bias, thereby potentially influencing effect estimates. Next, the study was conducted in a geographically specific area with a mild Mediterranean climate. It potentially facilitated more outdoor activity (despite the period of the study) and therefore limits the generalizability of findings. Additionally, PA was assessed using subjective reporting, which may be prone to recall bias or social desirability effects. Finally, PL was evaluated using a single self-report instrument (PLAYself), which may not fully capture the multidimensional nature of the construct. Therefore, further studies should apply different measurement tools in evidencing changes in PL in early school-age children. Also, a potential limitation of the present study is the increased risk of Type I error associated with the analysis of multiple outcomes, which may elevate the probability of false-positive findings. Finally, we did not examine individual responsiveness to the intervention. While previous research has shown that considerable inter-individual variability exists in training adaptations [77,78], future studies should consider analyzing responder profiles to better understand variability in PF outcomes.

Despite these limitations, the study also possesses some strengths. First, a wide range of PF tests we have used allowed a comprehensive evaluation of participants’ capacities, evidencing specific effects in different facets of PF. Second, the longitudinal design, incorporating baseline, post-intervention, and follow-up measurements, enabled the assessment of both immediate and retained effects of the intervention. Next, testing was conducted by experienced evaluators following standardized protocols, ensuring the consistency and reliability of data collection. Most importantly, the intervention was implemented in a real-world school setting, providing ecologically valid insights into the practical impact of PL-based education within the regular PE curriculum.

## 5. Conclusions

This study suggests small, but significant, improvements in specific PF indicators among children who participated in the PL-based intervention, specifically in abdominal strength and jumping power. These effects are likely due to the combination of structured and non-structured activities, empowered by PL-educational content that emphasized the importance, benefits, and accessibility of PA. The integration of video-based education and simple training strategies probably helped children to understand and apply fitness-related concepts in everyday contexts, which resulted in improvements of PF status.

In terms of PL, the study did not evidence substantial changes in the cognitive or affective domains, possibly due to the limitations of the measurement tool used (PLAYself). While PLAYself has demonstrated reliability, it may lack sensitivity to detect subtle gains in self-perceived competence and knowledge. In other words, it is possible that the lack of effects of the applied intervention on PA is actually a consequence of the applied measurement tool. This raises the need to explore more responsive instruments, such as CAPL-2, in future PL evaluations.

It is important to note that the applied intervention was “educational”, and not “training-based” in nature. It did not increase exercise volume or intensity, but focused on improving children’s knowledge, understanding, and awareness of PA and PF. The observed improvements suggest that even brief, structured educational content integrated into regular physical education classes can positively influence PF outcomes. These findings highlight the value of pedagogical strategies in promoting children’s health without the need for additional training time or resources.

Future research should consider longer interventions, more diverse assessment tools, and designs that account for classroom-level clustering. Including direct measures of motivation, knowledge, and environmental influences (e.g., family or community support) could provide a fuller understanding of PL’s impact. Multi-site trials would also strengthen generalizability and identify which children benefit most from these programs.

## Figures and Tables

**Table 1:** Main characteristics of the video contents.

Theme	Number of Videos	Key Topics Covered
Physical Literacy	2	Cognitive domain; affective domain
Cardiovascular Endurance	3	Cognitive and affective domains + practical examples
Strength	3	Cognitive and affective domains + practical examples
Flexibility	2	Cognitive and affective domains + specific examples
Healthy Habits and Nutrition	2	The significance of balanced nutrition and hydration (with regard to physical activity and health)

**Table 2:** Main characteristics of the program applied in experimental and control group.

Aspect	Experimental Group	Control Group
Number of PE Lessons	36 lessons over 12 weeks	36 lessons over 12 weeks
Curriculum Content	Standard PE + PL-focused educational content	Standard national PE curriculum
Educational Video Integration	12 videos shown 3 times each, with teacher-led discussion	No video content
Focus on Physical Literacy	Yes, emphasized throughout intervention	No targeted PL education
Use of Supplementary Materials	Yes, videos and practical tips provided	No Supplementary Materials
Instructional Time Adjustment	5–7 min per lesson substituted standard PL education	No instructional time reallocation
Assessment	Yes (initial–final–follow-up)	Yes (initial–final–follow-up)

**Table 3:** Main and interaction effects for the factorial multivariate analysis of variance for repeated measurements calculated for the set of physical fitness variables (F-test) with partial eta squared (η^2^).

	Intercept	Main Effects			Interaction Effects			
		Time	Gender	Group	Time × Gender	Time × Group	Group × Gender	Time × Gender × Group
F-test	2615 *	1391 *	1.06	1.78	2.44	2.98 *	2.23	2.21
η^2^	0.98	0.98	0.02	0.04	0.09	0.12	0.05	0.05

* denotes statistical significance of *p* < 0.05.

**Table 4:** Main and interaction effects of the repeated measures factorial ANOVA (F-test) with partial eta squared (η^2^).

		Intercept	Main Effects			Interaction Effects			
			Time	Gender	Group	Time × Gender	Time × Group	Group × Gender	Time × Gender × Group
Body height	F-test	4868 *	740.2 *	1.77	3.2	7.97 *	2.90	0.01	2.81
	η^2^	0.99	0.84	0.01	0.02	0.06	0.02	<0.01	0.02
Body mass	F-test	1807 *	16.52 *	1.36	3.25	0.77	0.87	1.33	1.21
	η^2^	0.93	0.10	<0.01	0.02	<0.01	<0.01	<0.01	<0.01
BMI	F-test	3113 *	9.89 *	0.93	1.81	1.45	0.35	1.31	0.05
	η^2^	0.96	0.07	<0.01	0.01	0.01	<0.01	0.01	<0.01
Broad jump	F-test	6202 *	19.07 *	0.85	0.01	1.12	3.26 *	2.13	1.25
	η^2^	0.97	0.12	<0.01	<0.01	<0.01	0.02	0.01	<0.01
Push-ups	F-test	211*	11.24*	3.35	2.93	0.86	2.91	0.08	5.04 *
	η^2^	0.6	0.07	0.02	0.02	<0.01	0.02	<0.01	0.04
Sit-ups	F-test	409.21 *	48.34 *	0.38	0.11	2.77	5.92 *	0.24	1.86
	η^2^	0.75	0.26	<0.01	<0.01	0.02	0.04	<0.01	0.01
Torso lift	F-test	2341 *	11.19 *	1.97	1.13	0.39	0.91	0.45	0.25
	η^2^	0.94	0.07	0.01	<0.01	<0.01	<0.01	<0.01	<0.01
Sit-and-reach	F-test	3206 *	28.29 *	41.25 *	0.02	1.94	2.23	0.67	0.91
	η^2^	0.95	0.17	0.24	<0.01	0.01	0.01	<0.01	<0.01
Multistage fitness test	F-test	1184*	4.81*	15.39*	0.02	0.39	1.01	0.33	0.37
	η^2^	0.89	0.04	0.1	<0.01	<0.01	<0.01	<0.01	<0.01
Physical literacy	F-test	6244 *	21.22 *	0.01	1.9	0.15	0.21	0.16	2.01
	η^2^	0.97	0.13	<0.01	0.01	<0.01	<0.01	<0.01	0.01
Physical activity	F-test	4547 *	3.87 *	10.85 *	1.04	0.11	0.39	2.47	2.12
	η^2^	0.97	0.03	0.07	<0.01	<0.01	<0.01	0.01	0.02

* denotes statistical significance of *p* < 0.05.

**Table 5:** Descriptive statistics for the total sample of participants and ANOVA post hoc differences.

	Initial	Final	Follow-up
	Mean ± SD	Mean ± SD	Mean ± SD
Body height (cm)	143.47 ± 7.31	144.79 ± 7.36 ^¥^	149.01 ± 8.11 ^¥,£^
Body mass (kg)	37.33 ± 10.48	38.14 ± 10.11 ^¥^	40.01 ± 10.6 ^¥,£^
BMI (kg/m^2^)	17.74 ± 3.41	19.01 ± 3.77 ^¥^	17.76 ± 4.02
Broad jump (cm)	140.64 ± 21.09	143.39 ± 21.14	146.28 ± 21.58 ^¥^
Push-ups (reps)	11.61 ± 9.51	10.41 ± 11.69	12.55 ± 11.21 ^£^
Sit-ups (reps)	15.8 ± 12.41	17.74 ± 12.34	24.61 ± 14.14 ^¥^
Torso lift (cm)	15.88 ± 4.51	17.78 ± 4.64 ^¥^	16.41 ± 5.29
Sit-and-reach (cm)	44.17 ± 11.42	48.27 ± 12.22 ^¥^	49.35 ± 11.82 ^¥^
Multistage fitness test (levels)	7.68 ± 2.62	8.99 ± 8.08	10.14 ± 11.58 ^¥^
Physical literacy (score)	67.24 ± 11.63	71.98 ± 11.6	72.23 ± 11.24 ^¥^
Physical activity (score)	3.41 ± 0.72	3.42 ± 0.72	3.55 ± 0.68 ^¥^

^¥^—significantly different from initial measurement, ^£^—significantly different from final measurement.

## Data Availability

The data presented in this study are available on request from the corresponding author due to the necessity of preserving the participants’ anonymity and project requirements.

## Supplementary Materials

The following supporting information can be downloaded at: https://www.mdpi.com/article/10.3390/healthcare14050695/s1, Figure S1: Percentage of changes in study variables during the study course for total sample (A), control group (B), and experimental group (C); The video materials of the applied intervention are available here: Video S1: https://tinyurl.com/videosPL (accessed on 8 January 2026).

## Abbreviations

The following abbreviations are used in this manuscript:

PF	Physical Fitness
PA	Physical Activity
PL	Physical Literacy
PE	Physical Education
