# Supplementary material for: Physical Literacy-Focused Education Improves Fitness Markers in Preadolescents: Implications for School-Based Health Promotion
Source: Healthcare (Basel). 2026 Mar 9;14(5):695. doi: 10.3390/healthcare14050695 (PMC12984826; doi:10.3390/healthcare14050695)
Supplement: Supplementary file 1 [file healthcare-14-00695-s001.zip › healthcare-4116245-supplementary.pdf]

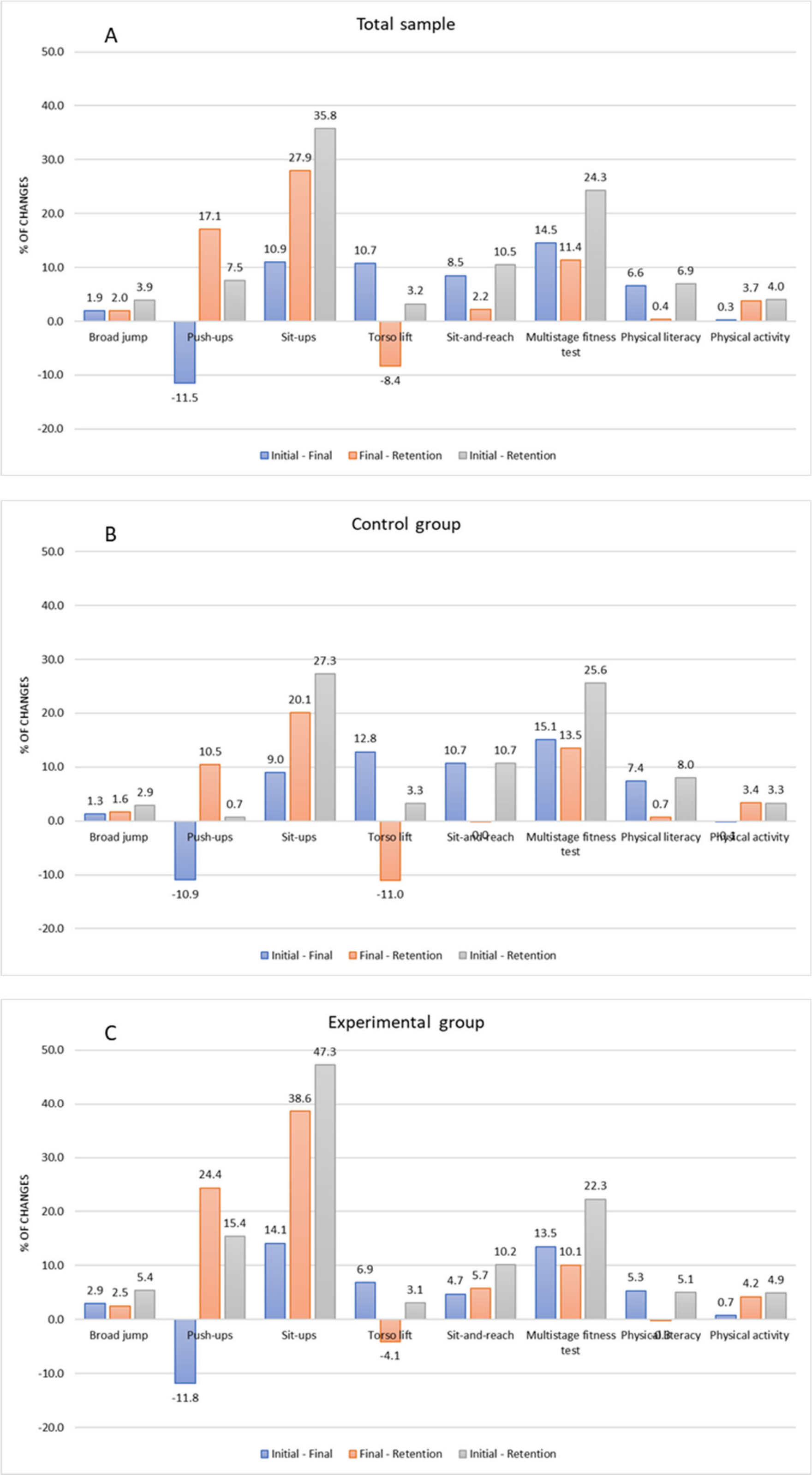


Figure S1. Percentage of changes in study variables during the study course for total sample (A), control group (B), and experimental group (C).
